# Association between blood lipids, BMI and gestational diabetes mellitus in early pregnancy: a clinical study and Mendelian randomization

**DOI:** 10.3389/fendo.2026.1870557

**Published:** 2026-08-07

**Authors:** Yukun Wang, Xinyu He, Lijie Nie, Chunyi Yue, Ruiqi Li, Lei Mo, Jian Huang, Xiangyuan Yu

**Affiliations:** 1 The Guangxi Key Laboratory of Environmental Exposomics and Entire Lifecycle Heath, School of Public Health, Guilin Medical University, Guilin, China; 2 Department of Obstetrics and Gynecology, The Second Affiliated Hospital of Guilin Medical University, Guilin, China; 3 Department of Cell and Genetics, School of Basic Medical Sciences, Guilin Medical University, Guilin, China; 4 Department of Epidemiology and Health Statistics, School of Public Health, Guilin Medical University, Guilin, China

**Keywords:** association, blood lipids, gestational diabetes mellitus, Mendelian randomization, nomogram

## Abstract

**Background:**

Gestational diabetes mellitus (GDM) women often have abnormal lipid levels during pregnancy. Moreover, lipid metabolism and body mass index (BMI) may influence the occurrence of GDM, and the potential relationship between them remains unclear.

**Objective:**

To explore the potential association between blood lipid levels, BMI and GDM in early pregnancy.

**Methods:**

Multivariate logistic regression analysis was employed, and the odds ratio (OR) and its 95% confidence interval (CI) were used to evaluate the associations between early pregnancy lipid levels, such as triglyceride (TG), total cholesterol (TC) as well as BMI and the risk of GDM. Based on the identified significant associated factors, a clinical prediction model was attempted to be constructed. Further use bidirectional Mendelian randomization (MR) analysis to evaluate the potential relationship between screened risk associated blood lipids and GDM.

**Results:**

In the case-control study, multivariate logistic regression analysis showed that BMI (OR = 1.12, 95% CI = 1.07-1.16, *P* < 0.05), TG (OR = 1.27, 95%CI=1.13-1.42, *P* < 0.05), TC (OR = 1.12, 95%CI=1.01-1.25, *P* < 0.05), LDL-c (OR = 1.13, 95%CI=1.01-1.27, *P* < 0.05) and HbA1c (OR = 5.94, 95%CI=4.28-8.25, *P* < 0.05) were significantly associated with the risk of GDM after adjusting for age. Based on TG, TC, LDL-c and BMI, the nomogram demonstrated AUCs of 0.772 in the training set, 0.754 in internal validation and 0.759 in external validation, indicating good and stable predictive performance. In the forward MR analysis, genetic evidence supported potential associations between TG (OR = 1.24, 95%CI=1.10-1.40, *P* < 0.05), BMI (OR = 1.75, 95%CI=1.51-2.03, *P* < 0.05) and GDM; while the reverse MR analysis indicated that GDM may have an association effect on TG (OR = 1.03, 95%CI=1.02-1.04, *P* < 0.05). Leave-one-out sensitivity analyses confirmed the robustness and reliability of the primary findings.

**Conclusion:**

This study provides suggestive evidence that TG and BMI may be associated with GDM risk, supported by clinical and genetic evidence. A nomogram demonstrated good and stable predictive performance. and MR analysis provided suggestive evidence of a potential bidirectional relationship between GDM and elevated TG. Further validation is needed.

## 1 Introduction

Gestational diabetes mellitus (GDM) is a metabolic disorder caused by carbohydrate intolerance during pregnancy, which poses a serious threat to the health of mothers and infants (1, 2). Globally, it affects approximately 9-25% of pregnancies and is showing a continuous upward trend (3). GDM not only increases the risk of complications such as gestational hypertension, premature birth, and cesarean section in pregnant women, but is also closely related to long-term metabolic diseases in offspring, such as obesity and abnormal glucose metabolism (4, 5). The etiology of GDM is complex and not fully understood, though insulin resistance and β-cell dysfunction are central to its pathogenesis.

Traditional research has mostly focused on glucose metabolism abnormalities in the middle and late stages of pregnancy, but insufficient attention has been paid to the mechanism of the association between metabolic disorders in early pregnancy and the occurrence and development of GDM. More and more evidence suggest that abnormal lipid metabolism plays an important role in the pathogenesis of GDM (6, 7). In clinical practice, early pregnancy is a critical period for embryonic organ development and maternal metabolic adaptation; abnormal elevation of blood lipid levels at this stage may contribute to GDM pathophysiology by inducing insulin resistance, impairing β-cell function, and triggering chronic inflammation (8, 9).

Experimental studies have established plausible mechanisms linking lipid excess to β-cell injury. Chronic exposure of pancreatic β-cells to elevated fatty acids impairs glucose-stimulated insulin secretion, induces islet inflammation and apoptosis through NF-κB activation, and promotes β-cell dedifferentiation (10). Additionally, saturated free fatty acids induce placental trophoblast lipoapoptosis via caspase 3/7 activation, which may compromise placental function and further exacerbate metabolic stress in pregnancy (11). Hypertriglyceridemia in early pregnancy has been associated with impaired insulin action and inadequate β-cell compensation, providing a mechanistic link between lipid dysregulation and subsequent GDM development (9).

Epidemiological studies have indicated that higher pre-pregnancy body mass index (BMI), and dyslipidemia such as high triglyceride (TG), low-density lipoprotein cholesterol (LDL-c), high-density lipoprotein cholesterol (HDL-c) are significantly associated with the risk of GDM (12–14). On the other hand, hyperlipidemia can damage placental endothelial function, elevate oxidative stress, and potentially affect the occurrence of GDM (15, 16). These pieces of evidence suggest that lipid metabolism plays a crucial role in the occurrence and development of GDM. However, there is still a lack of large-scale research in Guilin, China to verify the association relationship between early pregnancy blood lipid indicators and GDM incidence.

Mendelian randomization (MR) is a causal inference method based on genetic variation that can evaluate the association relationship between exposure factors and health outcomes, providing highly reliable causal evidence for medical research (17). This study uses hospital sourced case-control studies to clarify the association between early pregnancy blood lipid profiles (TG, TC, LDL-c, HDL-c), BMI and the occurrence of GDM, and further uses MR to explore the potential association direction, aiming to tentatively provide new theoretical basis and clinical strategies for early warning and etiological intervention of GDM.

## 2 Methods

### 2.1 Clinical data collection

According to the criteria of the International Association of Diabetes and Pregnancy Study Group (IADPSG) (18),the diagnostic criteria for GDM are as follows: During the 24th to 28th week of pregnancy, a 75 g oral glucose tolerance test (OGTT) is performed. GDM can be diagnosed based on at least one threshold value reached or exceeded in the OGTT: fasting blood glucose (FPG) ≥5.1 mmol/L, 1h blood glucose ≥10.0 mmol/L, or 2h blood glucose ≥8.5 mmol/L. Inclusion criteria required that participants have no familial relationships and be experiencing singleton pregnancy. Exclusion criteria included: (1) Presence of endocrine diseases; (2) Serious systemic diseases; (3) History of pre-pregnancy type 1 or type 2 diabetes; (4) Use of lipid-lowering agents or corticosteroids before or during early pregnancy;(5) Multiple pregnancies;(6) Chronic hypertension, untreated thyroid dysfunction, severe renal or hepatic disease; (7) Incomplete OGTT data;(8) Other pregnancy complications before pregnancy.

Data of 1,274 research subjects from the First Affiliated Hospital of Guilin Medical University, including 553 GDM and 721 normal pregnancies during the same period and was frequency-matched to cases by age and gestational week in a ratio of approximately 1:1.3 at sampling, including age, height, weight, SBP, DBP, FPG, OGTT 1h, OGTT 2h, HbA1c, TG, TC, LDL-c and HDL-c. After matching, baseline characteristics between the two groups were compared to assess the success of the matching procedure, as shown in **Table 1**. BMI calculation formula: Weight (kg)/Height (m)². All blood samples are collected in the morning after fasting for at least 8 hours during early pregnancy (6–12 weeks). All participants have signed informed consent forms and the research protocol has been approved by the Ethics Committee of Guilin Medical University (Number: GLMC20131205).

**Table 1:** Demographic and clinical characteristics.

Clinical data	GDM (n=553) mean ± SD	Controls (n=721) mean ± SD	*t*	*P*
Age (years old)	31.45 ± 4.73	28.88 ± 4.18	10.08	<0.001***
BMI (kg/m^2^)	23.08 ± 3.61	21.67 ± 3.06	7.39	<0.001***
TG (mmol/L)	2.67 ± 1.20	2.37 ± 1.01	4.83	<0.001***
TC (mmol/L)	5.38 ± 1.16	5.25 ± 1.07	2.06	0.040**
LDL-c (mmol/L)	3.49 ± 1.03	3.36 ± 1.04	2.16	0.030**
HDL-c (mmol/L)	1.66 ± 0.42	1.66 ± 0.41	0.01	0.990
SBP (mmHg)	111.52 ± 10.60	109.29 ± 9.45	3.96	<0.001***
DBP (mmHg)	70.44 ± 8.63	68.43 ± 8.11	4.27	<0.001***
FPG (mmol/L)	5.18 ± 1.13	4.41 ± 0.36	15.45	<0.001***
OGTT 1h (mmol/L)	9.71 ± 2.11	7.00 ± 1.42	26.07	<0.001***
OGTT 2h (mmol/L)	8.24 ± 1.91	6.10 ± 1.10	23.66	<0.001***
HbA1c (%)	5.42 ± 0.60	4.94 ± 0.67	13.18	<0.001***

TG, triglyceride; TC, total cholesterol; LDL-c, low-density lipoprotein cholesterol; HDL-c, high-density lipoprotein cholesterol; SBP, systolic blood pressure; DBP, diastolic blood pressure; FPG, fasting plasma glucose.

The *P*-values are derived from the two-sided independent sample t-test.

Significance: * *P* < 0.05; ** *P* < 0.01; ***P < 0.001.

Bold values: statistically significant.

Multivariate logistic regression analysis was performed to estimate the association between blood lipids and BMI, and the risk of GDM, and the odds ratios (ORs) and their 95% confidence intervals (CIs) were calculated to quantify the strength of association. A two-sided *P* < 0.05 was considered statistically significant. A nomogram was constructed using the R package ‘rms’ based on the corresponding regression coefficients, the area under the receiver operating characteristic curve (AUC) was used to evaluate the model’s performance. The training set and internal validation set were randomly divided in a 7:3 ratio. The calibration plots were generated using internal validation with a bootstrap method involving 1000 resamples to evaluate the consistency between the predicted and observed values. Besides, decision curve analysis (DCA) was also conducted to evaluate the clinical effectiveness and benefit of the nomogram. For external validation, an independent cohort of 390 participants (180 GDM and 210 controls) was collected from the Second Affiliated Hospital of Guilin Medical University during a different time period. The same inclusion and exclusion criteria were applied. The established nomogram was applied to this external cohort to evaluate its discriminative performance using the AUC, calibration plots, and DCA. The events per variable (EPV) ratio was calculated to assess the adequacy of sample size for model validation. With 5 predictors in the nomogram, the EPV was 110.6 for the internal validation set (553 GDM cases) and 36 for the external validation set (180 GDM cases), both exceeding the recommended minimum of 10 (19).

### 2.2 Mendelian randomization analysis

#### 2.2.1 Study design

This study was designed and reported according to the STROBE-MR guidelines (20), utilizing a bidirectional two-sample MR approach to analyze the potential association relationship between four metabolic risk factors blood lipids, BMI and GDM. In investigating the association relationship between exposures and outcomes, single nucleotide polymorphisms (SNPs) were selected as instrumental variables (IVs). All chosen IVs must meet the following three core assumptions: (1) Relevance assumption: IVs must be strongly associated with the exposure; (2) Exclusivity assumption: IVs should affect the outcome solely through the exposure and not through other pathways; (3) Independence assumption: IVs must be independent of any known or unknown confounders.

#### 2.2.2 Data sources

The GWAS summary data used in this study include: TG (ID: ieu-b-111), TC (ID: ebi-a-GCST90025953), LDL-c (ID: ebi-a-GCST90101745), HDL-c (ID: ebi-a-GCST90101746), BMI (ID: ebi-a-GCST90013870) and GDM (ID: finn-b-GEST_DIABETES). The GDM GWAS data were obtained from the FinnGen consortium via the IEU OpenGWAS platform, including 5,687 GDM and 117,892 controls of European ancestry. According to the FinnGen endpoint definitions, GDM cases were identified using hospital discharge records with ICD-10 code O24.4. All data are available through the IEU OpenGWAS project (https://gwas.mrcieu.ac.uk/).

The data with the ebi prefix provided by the European Bioinformatics Institute, the ieu data are from the Integrative Epidemiology Unit, and the finn data are sourced from the FinnGen consortium. Detailed information about dataset can be found in the **Table 2**. All the datasets used are from public databases and there are no ethical review issues.

**Table 2:** Mendelian Randomization Analysis: information of datasets.

Trait	nSNP	Population	Year	ID
TG	12321875	European	2020	ieu-b-111
TC	4232052	European	2021	ebi-a-GCST90025953
LDL-c	21372748	Sub-Saharan African	2022	ebi-a-GCST90101745
HDL-c	21361416	Sub-Saharan African	2022	ebi-a-GCST90101746
BMI	10783680	European	2021	ebi-a-GCST90013870
GDM	16379784	European	2021	finn-b-GEST_DIABETES

TG, triglyceride; TC, total cholesterol; LDL-c, low-density lipoprotein cholesterol; HDL-c, high-density lipoprotein cholesterol; BMI, body mass index; GDM, gestational diabetes mellitus.

#### 2.2.3 Instrument variables selection

To satisfy the three core assumptions of instrumental variables, the following selection criteria were implemented: (1) Genome-wide significance was established at a stringent threshold of *P* < 5×10^-8^; (2) Independence was ensured by applying a linkage disequilibrium (LD) threshold of r^2^<0.001 within 10,000 kb clumping window; (3) Instrument strength was evaluated using the F calculated for each SNP. The proportion of variance explained (R²) was calculated for F-statistic computation using the standard formula. IVs with F<10 were regard as weak instruments in subsequent analyses (21, 22). No independent R² threshold was applied for variant selection.

(1) $${\text{R}}^{2}=2\times (1-\text{EAF})\times \text{EAF}\times {\text{β}}^{2}$$

(2) $$\text{F}=\frac{{\text{R}}^{2}\times (\text{n}-1-\text{k})}{(1-{\text{R}}^{2})\times \text{k}}$$

#### 2.2.4 Analytical methods

Inverse variance weighted random-effects method was used as the primary approach for MR analysis (Equation 1). The validity of the results assumes the absence of horizontal pleiotropy. To further assess the robustness of the primary inverse variance weighted (IVW) estimates, additional methods such as MR-Egger, maximum likelihood, weighted median, and weighted mode were employed (23, 24). In the presence of horizontal pleiotropy, the MR-Egger intercept is used to evaluate this potential bias (Equation 2). If the intercept is equal to zero, it indicates no horizontal pleiotropy, consistent with the IVW (23).

The robustness of the MR estimates was assessed when the results from multiple MR methods were consistent (23). Cochran’s Q test was used to evaluate heterogeneity among the IV, while MR-PRESSO and MR-Egger intercept analyses were employed to test for pleiotropy (23, 25). Leave-one-out sensitivity analysis was performed, systematically removing each unique IV to assess the impact on the MR association (26).

Prior to MR analysis, SNPs with incompatible alleles were removed. Additionally, palindromic SNPs with intermediate allele frequencies were excluded to avoid strand ambiguity. A total of 76 SNPs were removed in the forward MR analysis and 4 SNPs in the reverse MR analysis. Detailed information on removed SNPs is provided in **Supplementary Table 1**.

The data were processed using R software (v4.4.0). MR Analysis was performed using the “TwoSampleMR” software package and the “MendelianRandomization” software package, statistical significance was defined as a two-sided *P* < 0.05.

## 3 Results

### 3.1 Clinical data analysis

**Figure 1:**
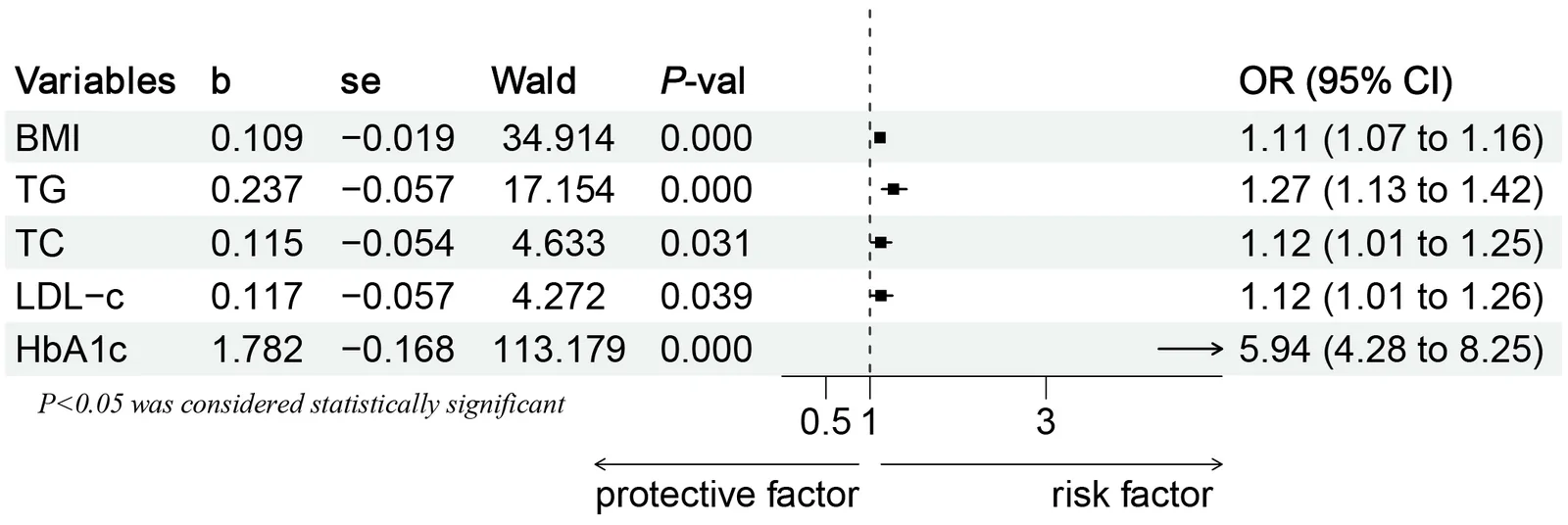
Clinical data analysis: multivariate logistic regression with GDM as the dependent variable. Logistic regression adjusted for age; Data are presented as ORs with 95%CI); A two-sided *P* < 0.05 was considered statistically significant.

**Figure 2:**
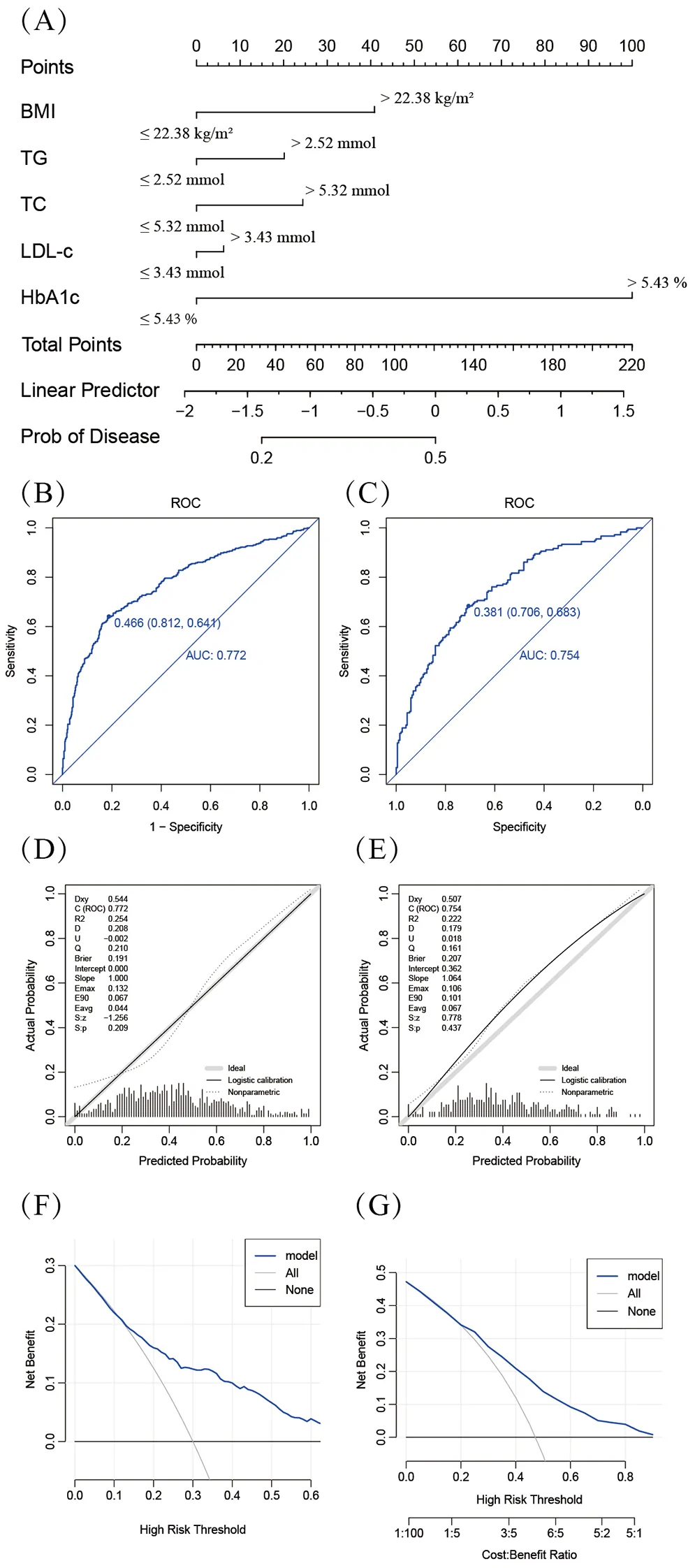
The development and internal validation of a nomogram model for predicting GDM risk. **(A)** A nomogram model based on logistic regression for BMI, TG, TC, LDL-c and HbA1c. **(B, C)** ROC curves for training set and validation. **(D, E)** Calibration plots for training set and validation. **(F, G)** A decision curve analysis (DCA) for training set and validation.

The characteristics of participants are shown in **Table 1**. Clinical indicators in GDM differed from those in the controls, including age, BMI, SBP, DBP, TG, LDL-c, FPG, OGTT 1h, OGTT 2h and HbA1c (*P* < 0.001). Multivariate logistic regression analysis was performed to identify independent risk factors for GDM. After adjusting for age, BMI (OR = 1.12, 95% CI = 1.07-1.16, *P* < 0.05, TG (OR = 1.27, 95%CI=1.13-1.42, *P* < 0.05), TC (OR = 1.12, 95%CI=1.01-1.25, *P* < 0.05), LDL-c (OR = 1.13, 95%CI=1.01-1.27, *P* < 0.05) and HbA1c (OR = 5.94, 95%CI=4.28-8.25, *P* < 0.05) were significantly associated with the increasing risk of GDM as shown in **Figure 1**. Based on the results of multivariate logistic regression, a nomogram was developed to graphically represent the combined contribution of BMI, TG, TC, LDL-c and HbA1c to GDM risk, the AUC was 0.772 in the training set and 0.754 in the internal validation set, suggesting good and stable predictive performance (**Figures 2B, C**). As shown in **Figures 2D, E**, the calibration plots showed good agreement between predicted and observed probabilities in both sets. DCA indicated that the model provided net clinical benefit across a range of threshold probabilities (**Figures 2F, G**).

**Figure 3:**
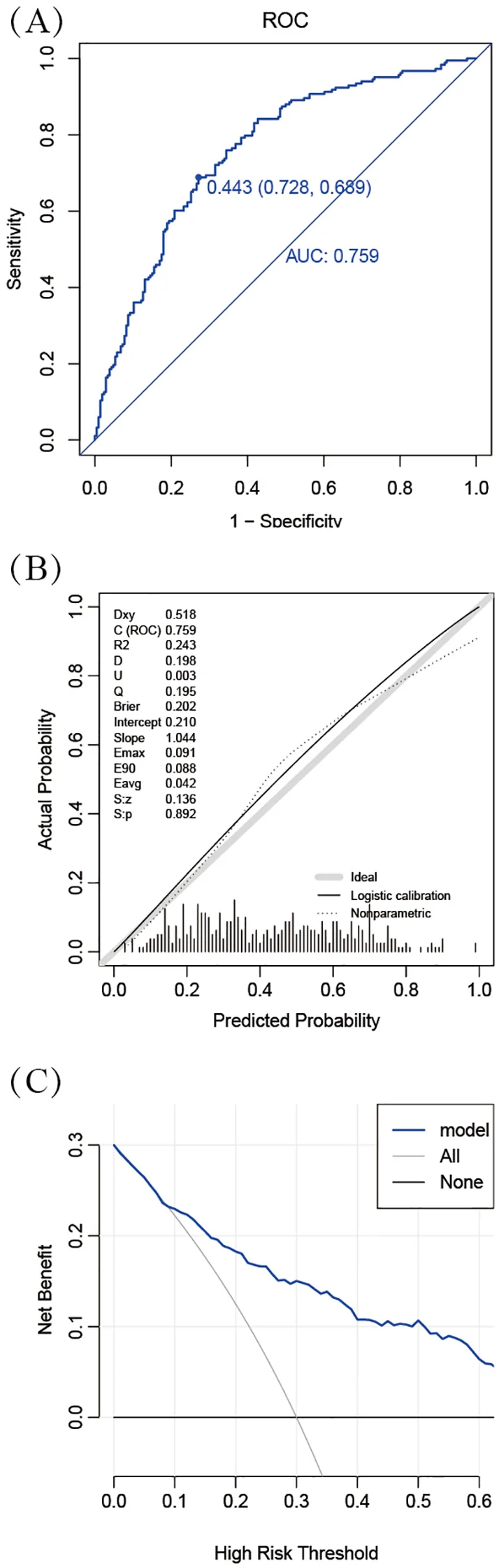
The external validation of a nomogram model for predicting GDM risk. **(A)** ROC curves for external validation. **(B)** Calibration plots for external validation. **(C)** DCA for external validation.

To further assess the generalizability of the nomogram, we performed external validation using an independent dataset (180 GDM and 210 controls) from the Second Affiliated Hospital of Guilin Medical University. The model achieved an AUC of 0.759, indicating acceptable performance in an external population. Calibration curves and DCA in the external cohort showed moderate agreement and net benefit within limited threshold ranges (**Figure 3**).

### 3.2 Forward MR analysis

**Figure 4:**
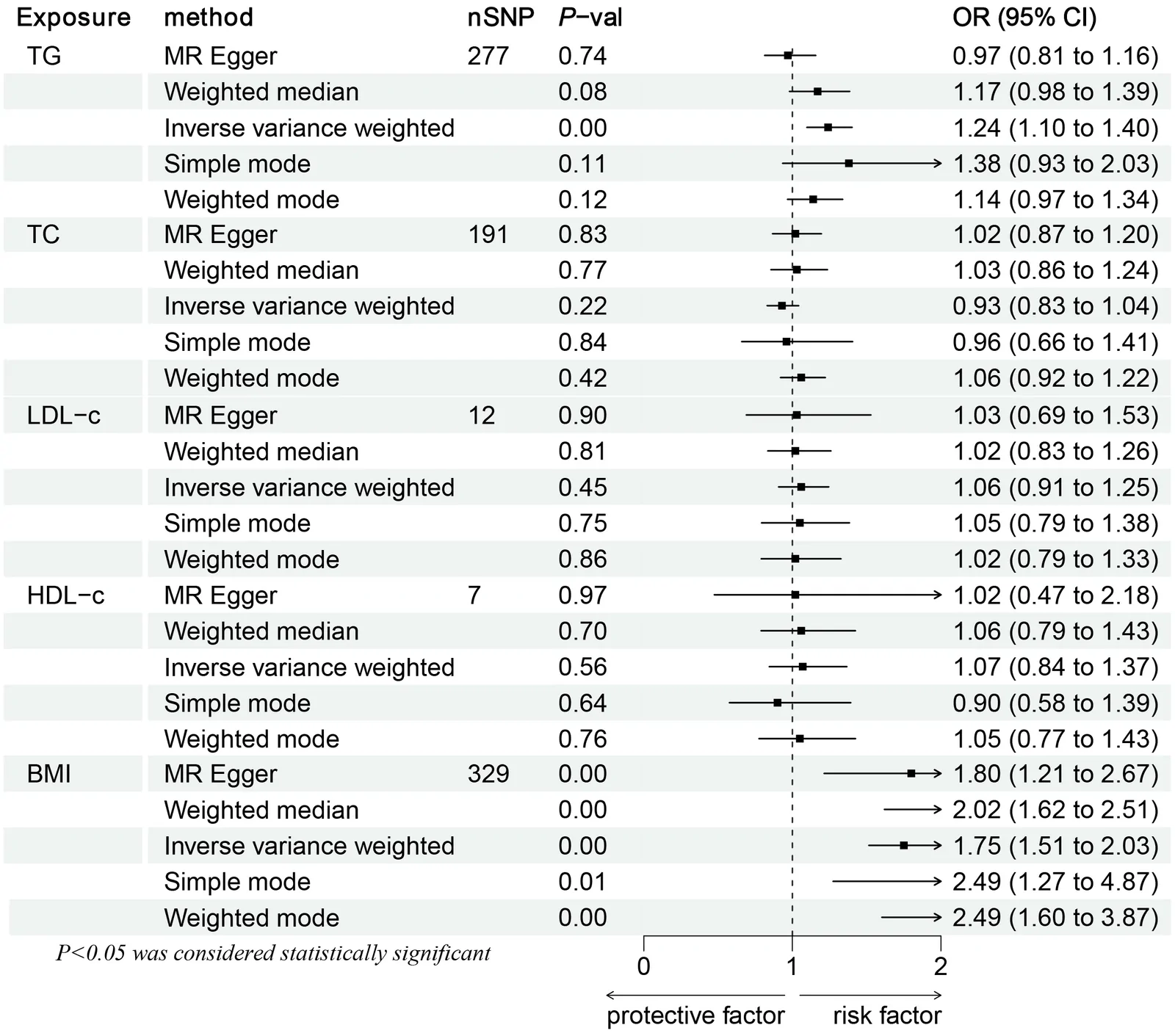
Forest plot of the MR analysis results on the impact of lipid indicators and BMI on GDM. Estimates were derived using the random-effects IVW method. The number of IVs (SNPs) for each exposure: TG (n=277), TC (n=191), LDL-c (n=12), HDL-c (n=7), and BMI (n=329); A two-sided *P* < 0.05 was considered statistically significant; No additional covariates were adjusted beyond the original GWAS models.

**Figure 5:**
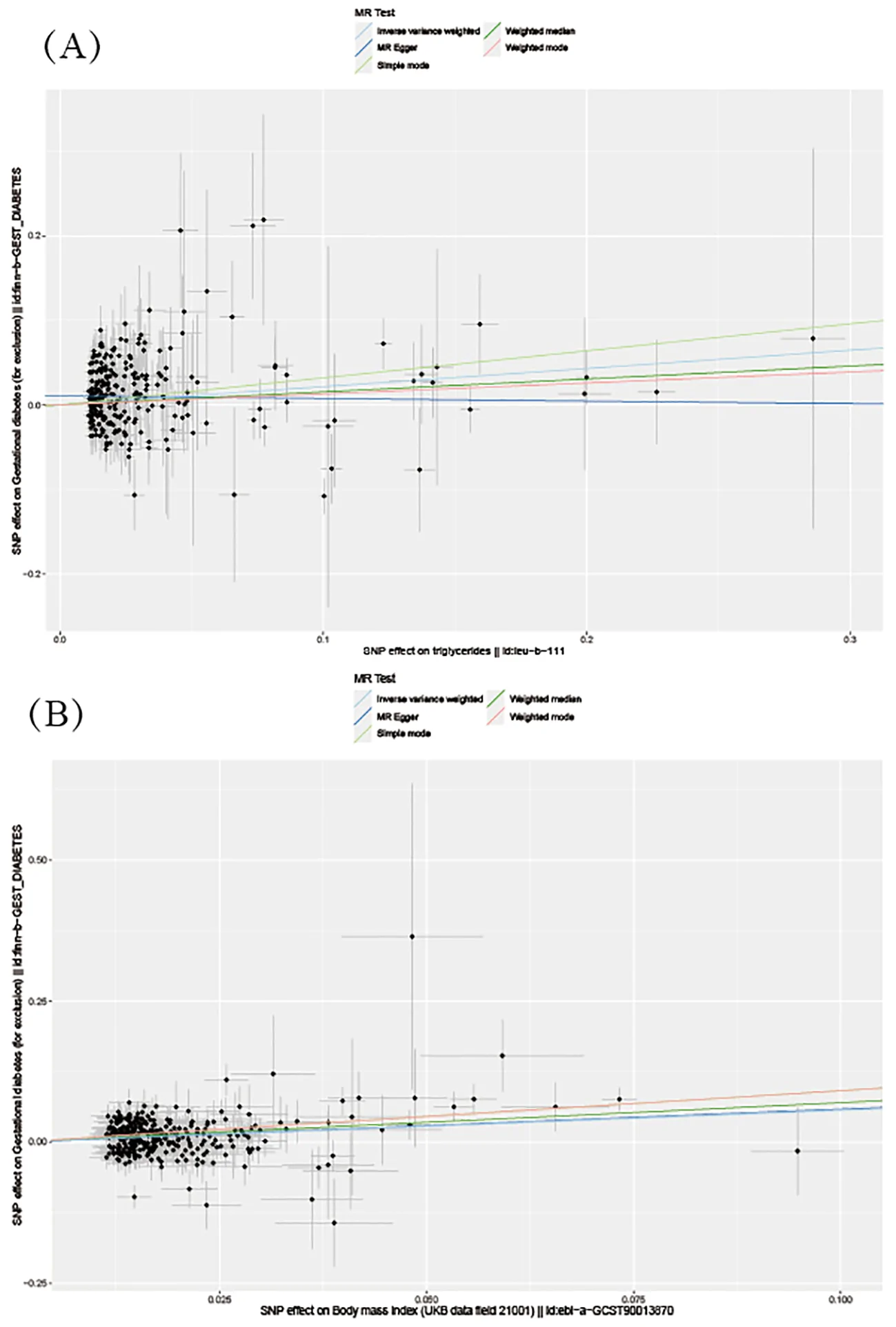
Scatter plot of the MR analysis results on the impact of TG **(A)** and BMI **(B)** on GDM. Each point represents a single SNP used as an IV; Slopes of the fitted lines correspond to MR estimates from different methods: IVW (light blue), MR-Egger (dark blue), simple mode (light green), weighted median (dark green) and weighted mode (red). The number of SNPs included: TG (n=277), BMI (n=329).

In the forward MR analysis, it showed that TG (OR = 1.24, 95%CI=1.10-1.40, *P* < 0.05) and BMI (OR = 1.75, 95%CI=1.51-2.03, *P* < 0.05) as risk factors showed suggestive associations with GDM under the IVW random-effect model. While there was no statistically significant association observed between TC (OR = 0.93, 95%CI=0.83-1.04, *P* = 0.22), LDL-c (OR = 1.06, 95%CI=0.91-1.25, *P* = 0.45), HDL-c (OR = 1.07, 95%CI= 0.84-1.37, *P* = 0.56) and GDM. As shown in **Figures 4**, **5** and **Supplementary Figure 1**.

### 3.3 Reverse MR analysis

**Figure 6:**
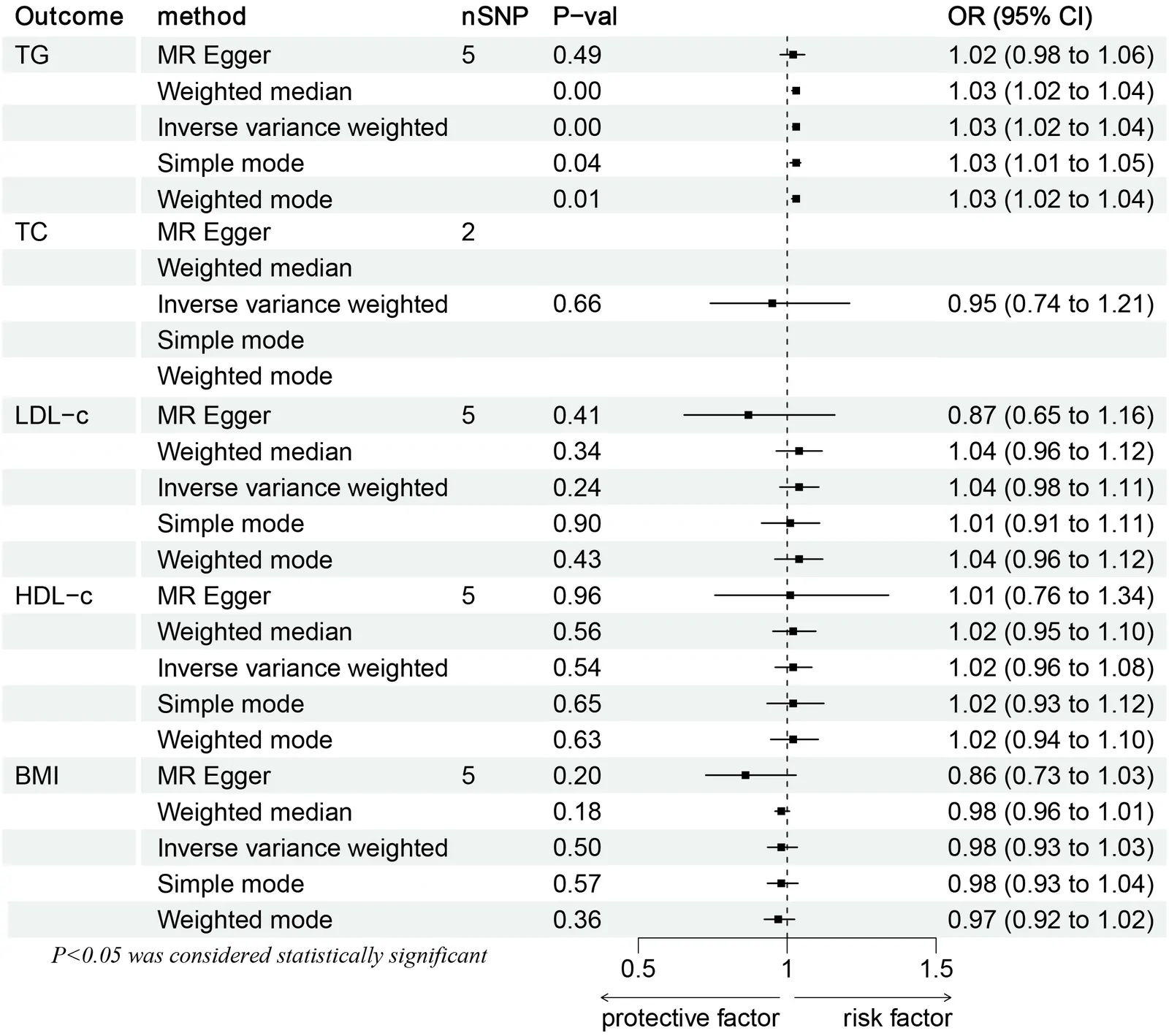
Forest plot of the MR analysis results on the impact of GDM on lipid indicators and BMI. Estimates were derived using the random-effects IVW method. The number of instrumental variables (SNPs) for each exposure: TG (n=5), TC (n=2), LDL-c (n=5), HDL-c (n=5), and BMI (n=5); A two-sided *P* < 0.05 was considered statistically significant; No additional covariates were adjusted beyond the original GWAS models.

**Figure 7:**
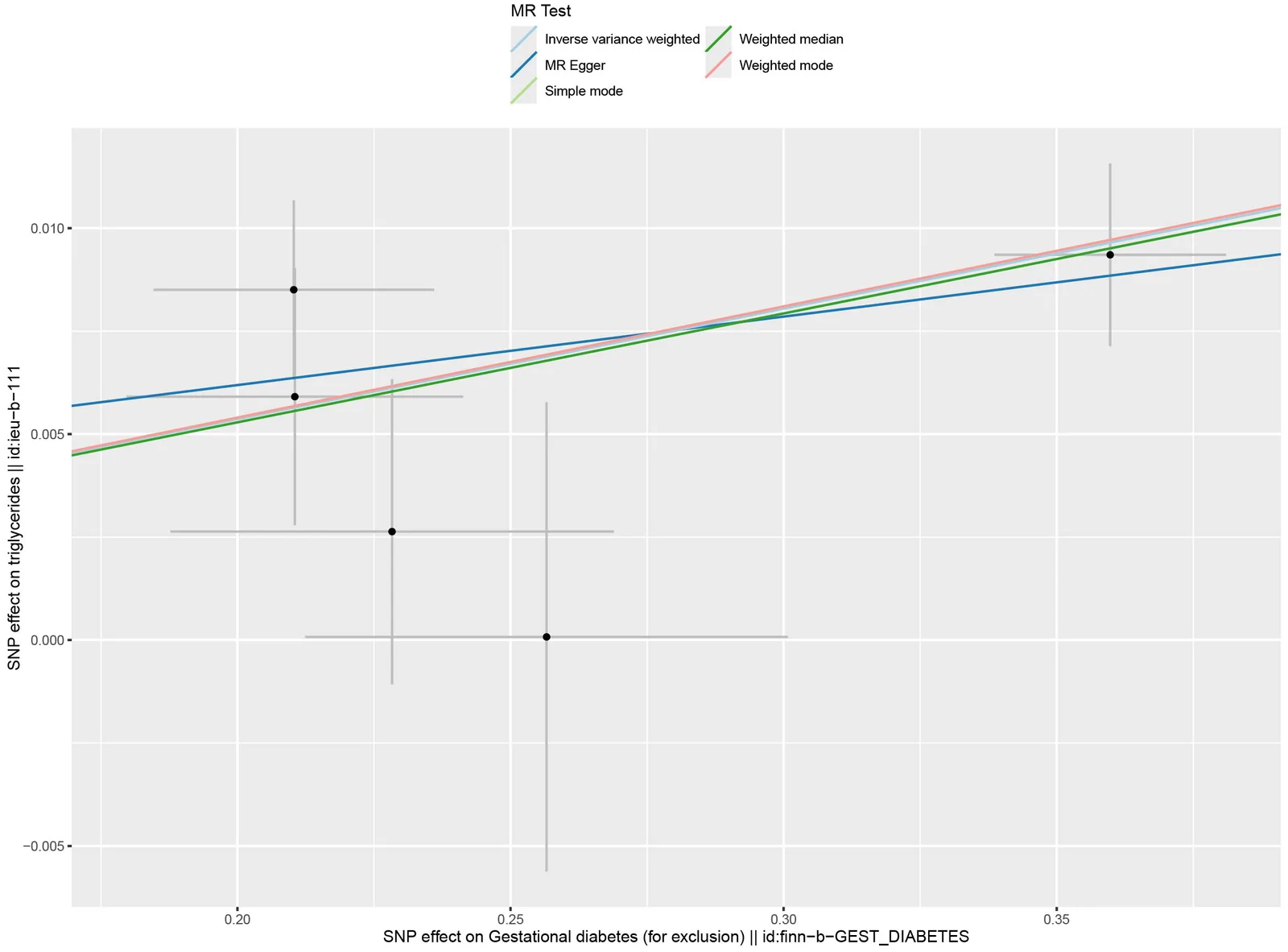
Scatter plot of the MR analysis results on the impact of GDM on TG. Each point represents a single SNP used as an IV. Each point represents a single SNP used as an IV; Slopes of the fitted lines correspond to MR estimates from different methods: IVW (light blue), MR-Egger (dark blue), simple mode (light green), weighted median (dark green) and weighted mode (red). The number of SNPs included: TG (n=5).

In the reverse MR analysis, GDM showed a significant association with TG under the IVW random-effect model as shown in **Figures 6**, **7** (OR = 1.03, 95%CI=1.02-1.04, *P* < 0.05). GDM had no significant association effects on LDL-c (OR = 1.04, 95%CI=0.98-1.11, *P* = 0.24), HDL-c (OR = 1.02, 95%CI=0.96-1.08, *P* = 0.54) and BMI (OR = 0.98, 95%CI=0.93-1.03, *P* = 0.50) (**Supplementary Figure 2**).

### 3.4 Sensitivity analysis

**Figure 8:**
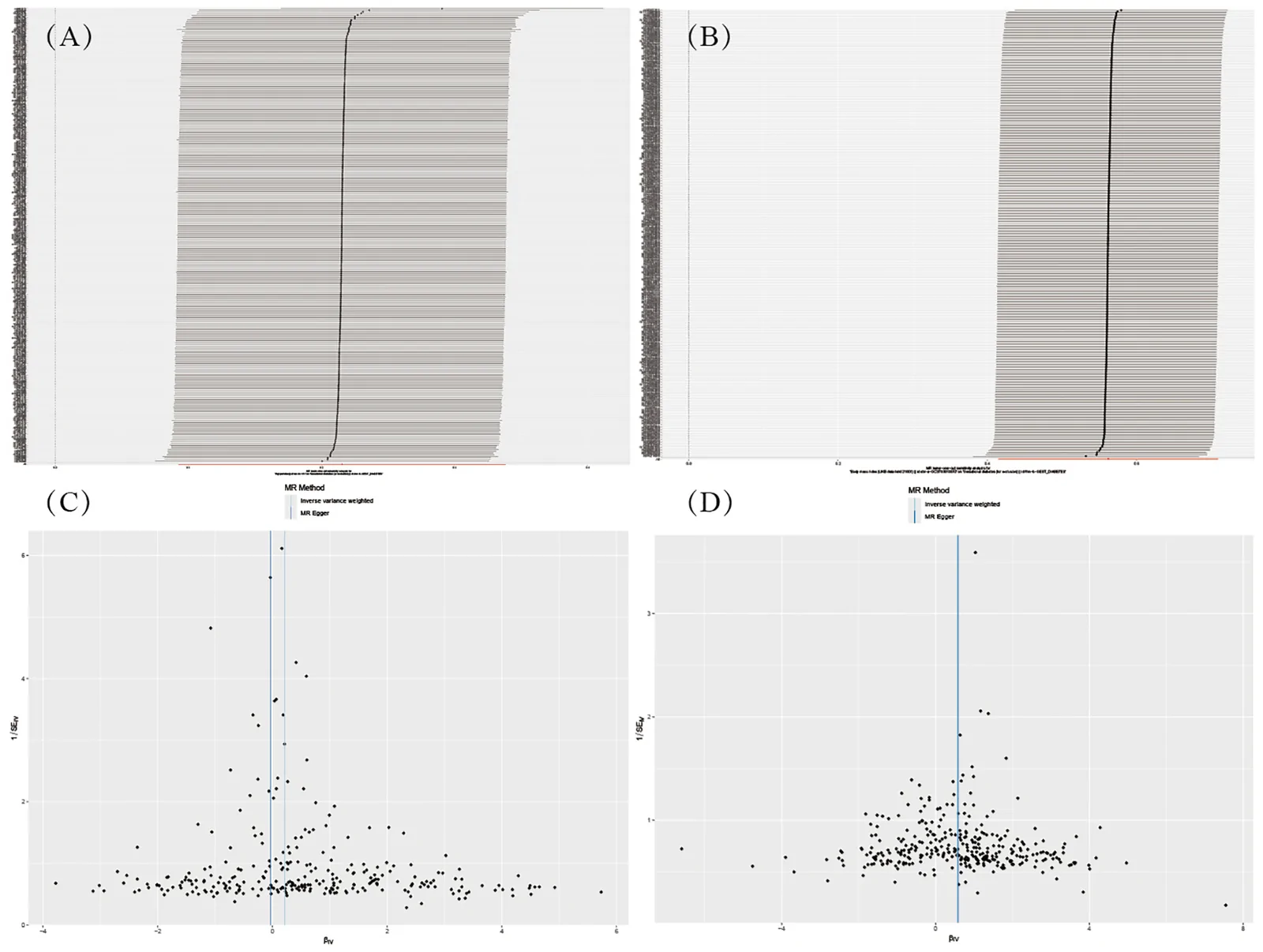
The leave-one-out plots of GDM and TG **(A)** and BMI **(B)** respectively. The red lines indicate the overall IVW estimates. The number of IVs (SNPs) for each exposure: TG (n=277) and BMI (n=329); The funnel plot of GDM and TG **(C)** and BMI **(D)** respectively. Approximate symmetry suggests no substantial directional pleiotropy.

Sensitivity analysis was conducted to assess the robustness of the significant results in the bidirectional MR. Forward MR analysis revealed that BMI showed a suggestive association with GDM, and no horizontal pleiotropy was found, supporting the reliability of the association relationship. However, association between TG and GDM was significant, its reliability is questionable due to significant heterogeneity and horizontal pleiotropy (**Table 3**). Leave-one-out analysis shows that the effect sizes of TG and BMI remained stable in the pooled effect size estimates after excluding any single SNP, with no change in direction, indicating that the conclusions do not depend on any specific SNP and the results are robust, funnel plots for these exposures demonstrated approximate symmetry, suggesting no substantial directional pleiotropy (**Figure 8**).

**Table 3:** Sensitivity analysis of exposure factors: lipid indicators, BMI.

Exposure Factor	Methods	Heterogeneity Test		Pleiotropy Test		
		Q	Q-*P*^a^	Egger_intercept	se	Egger-*P*^b^
TC	MR-Egger	244.255	0.004**	-0.005	0.003	0.126
	IVW	247.300	0.003**			
TG	MR-Egger	438.156	<0.001***	0.010	0.003	<0.001***
	IVW	459.992	<0.001***			
LDL-c	MR-Egger	7.048	0.721	0.004	0.019	0.858
	IVW	7.082	0.792			
HDL-c	MR-Egger	4.710	0.452	0.004	0.030	0.889
	IVW	4.732	0.579			
BMI	MR-Egger	399.312	0.004**	-0.001	0.004	0.889
	IVW	399.336	0.004**			

IVW. inverse variance weighted.

^a^ Significant heterogeneity: Q-*P* < 0.05; Significant horizontal pleiotropy: Egger-*P* < 0.05.

Significance: * *P* < 0.05; ** *P* < 0.01; ***P < 0.001.

Bold values: statistically significant.

**Figure 9:**
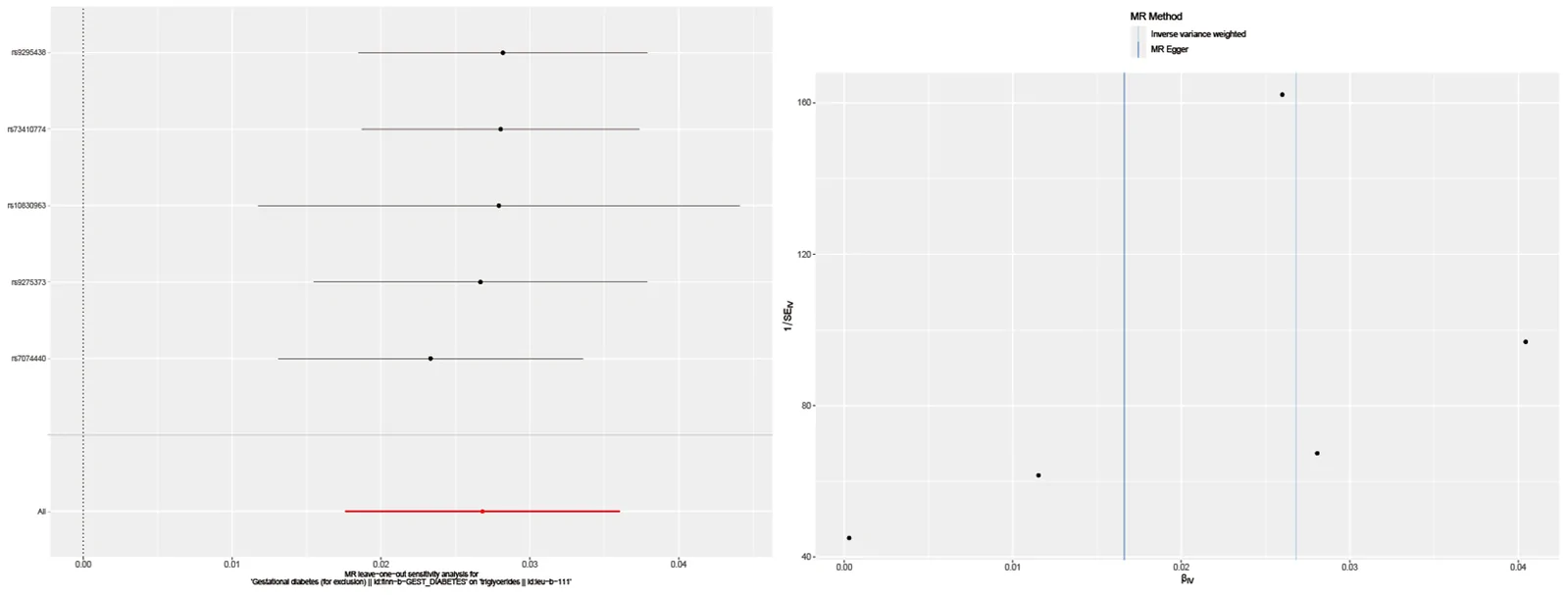
The leave-one-out plots of TG and GDM respectively. The red lines indicate the overall IVW estimates. The number of IVs (SNPs) for each exposure: TG (n=5); The funnel plot of TG and GDM respectively. Approximate symmetry suggests no substantial directional pleiotropy.

In the reverse MR analysis, GDM was associated with elevated TG levels. This association exhibited no heterogeneity or pleiotropy interference, demonstrating high robustness. The leave-one-out analysis showed that no single SNP disproportionately drove the overall estimate and the funnel plot demonstrated approximate symmetry, suggesting no substantial directional pleiotropy (**Figure 9**). No significant association effects were observed for LDL-c, HDL-c and BMI, and sensitivity analyses suggested no evidence of pleiotropy or heterogeneity (**Table 4**). Due to an insufficient number of valid instrumental variables after standard quality control (F<3), the reverse MR analysis for GDM on TC could not be conducted.

**Table 4:** Sensitivity analysis of exposure factors: GDM.

Outcome Factor	Methods	Heterogeneity Test		Pleiotropy Test		
		Q	Q-*P*^a^	Egger_intercept	se	Egger-Pb
TC	MR-Egger	——	——	——	——	——
	IVW	——	——			
TG	MR-Egger	3.764	0.288	0.003	0.006	0.650
	IVW	4.082	0.395			
LDL-c	MR-Egger	1.660	0.646	0.037	0.030	0.305
	IVW	3.187	0.646			
HDL-c	MR-Egger	1.186	0.756	0.002	0.030	0.938
	IVW	1.193	0.879			
BMI	MR-Egger	33.312	<0.001***	0.025	0.017	0.235
	IVW	57.690	<0.001***			

IVW, inverse variance weighted.

^a^ Significant heterogeneity: Q-*P* < 0.05; Significant horizontal pleiotropy: Egger-*P* < 0.05.

Significance: * *P* < 0.05; ** *P* < 0.01; ***P < 0.001.

Bold values: statistically significant.

Analyses for TC in reverse MR were not performed due to insufficient valid IVs (F<3).

## 4 Discussion

This study systematically explored the associations between early-pregnancy lipid profiles and GDM risk by integrating a clinical case-control study with bidirectional MR analysis. In the clinical analysis, after adjusting for age, we found that BMI, TG, TC, LDL-c and HbA1c were significantly associated with an increased risk of GDM, and a nomogram incorporating BMI, TG, TC, LDL-c and HbA1c showed good and stable predictive performance in both internal and external validation, with AUCs of 0.772 in the training set, 0.754 in internal validation, and 0.759 in external validation (390 participants from a different hospital). Notably, the clinical findings were not entirely consistent with the MR results. In forward MR analysis, genetically predicted elevated TG and increased BMI showed suggestive associations with GDM, Reverse MR analysis further indicated that GDM showed a suggestive effect on subsequent elevated TG levels, suggesting a potential bidirectional relationship between the two. Sensitivity analyses supported the robustness of the primary findings.

While both clinical and MR analyses provide suggestive evidence a positive association between TG and GDM, TC and LDL-c showed significant associations in the clinical model but not in the MR analysis. Several factors may explain this discrepancy. First, the clinical analysis is observational and subject to residual confounding, while we adjusted for age in the logistic regression, residual confounding from unmeasured lifestyle factors—such as pre-pregnancy diet, physical activity, and socioeconomic status—may have inflated the clinical associations for TC and LDL-c. MR analysis, by virtue of random assortment of genetic variants at conception, is largely unaffected by such confounding. Second, the clinical measurements reflect a single time point (6–12 weeks of gestation), A single early-pregnancy measurement may not capture the dynamic lipid trajectory that is biologically relevant to GDM development, potentially leading to misclassification of individual lipid exposure (27), while MR captures lifelong genetically determined lipid levels, which may not fully reflect pregnancy-related changes. Third, the MR analyses for TC and LDL-c may have been underpowered due to the limited number of valid instrumental variables or the relatively modest effect sizes of these lipid traits on GDM in European-ancestry GWAS. Finally, the ancestral mismatch between our Chinese clinical cohort and European GWAS data may have further attenuated the MR estimates for TC and LDL-c, as genetic effect sizes for lipid traits have been shown to vary across populations.

This study provides a comprehensive assessment of the preliminary association roles of multiple lipid markers in GDM development. Elevated TG levels were significantly associated with an increased risk of GDM. This finding is consistent with previous observational studies (28–30), and through the application of genetic instrumental variables, we further obtained genetic evidence supporting a potential association. Despite the heterogeneity, this may stem from TG metabolism being regulated by multiple environmental factors (31), with its genetic instrumental variables potentially influencing GDM risk through various biological pathways. The leave-one-out analysis revealed no directional change in the pooled effect size, and also failed to detect significant horizontal multi-effect bias. Therefore, the overall suggestive evidence regarding TG as a risk factor for GDM is supported by leave-one-out analysis, though caution is warranted due to heterogeneity. The positive association between BMI and GDM exhibits significant heterogeneity, which may reflect the diverse mechanisms through which obesity influences GDM risk (32, 33). When considering the combined effects of TG and BMI, overall obesity (represented by BMI) showed a suggestive independent association with GDM risk, with the mechanism potentially involving shared pathways. Elevated BMI is often accompanied by increased TG, and TG directly contributes to the pathophysiology of GDM by inducing lipotoxicity and further impairing insulin signaling (34, 35).

The long-term effects of GDM on lipid levels and BMI were not supported by the reverse MR analysis. This result may stem from differences that MR analysis captures lifelong exposures rather than pregnancy-specific longitudinal changes, whereas glucose and lipid metabolism is a long-term process. More complex mechanisms may exist between BMI, lipid metabolism, and GDM, requiring further validation through in-depth trials. These findings not only deepen our understanding of the pathogenesis of GDM but also provide important references for developing differentiated prevention strategies. Notably, the suggestive bidirectional relationship between GDM and elevated TG is supported by MR analysis, highlighting the central role of TG abnormalities throughout the course of GDM.

Given the observed associations between elevated TG, increased BMI with GDM risk, early identification of these modifiable factors could enable targeted lifestyle and pharmacological interventions before the onset of glucose intolerance (36). For instance, dietary modifications, adequate sleep, physical activity and lipid-lowering strategies could be considered for pregnant individuals with high TG, particularly those with elevated BMI (37, 38). Such a proactive approach may not only reduce GDM incidence but also mitigate subsequent maternal and fetal complications.

Despite the comprehensive nature of study, which incorporated diverse lipid profiles and body composition indices, several limitations should be considered. Since the publicly available GWAS data available is only from the European population, which a critical limitation is the ancestral mismatch between the European GWAS data and our Chinese clinical cohort. Quantitative evidence from trans-ethnic genetic correlation analyses suggests that while most lipid-associated genetic architectures are shared between European and East Asian populations, incomplete sharing exists (e.g., r_gen_ for LDL-c=0.778 between Europeans and Chinese). Approximately 75% of major lipid loci from European studies are reproducible in Asian populations, indicating that population-specific effects cannot be excluded (39). These differences may introduce bias and limit the generalizability of our MR findings to Chinese pregnant women. Future MR studies using large-scale Chinese-specific GWAS data are urgently needed to validate our results. Additionally, the clinical analysis relied on single-point lipid measurements obtained during early pregnancy, which may not fully capture the dynamic changes in lipid metabolism throughout gestation. Future prospective studies with serial lipid profiling are needed to clarify whether longitudinal lipid trajectories provide stronger and more consistent predictive value for GDM than single measurements. Although external validation was performed (n=390, from a different hospital), the sample size was modest, and larger multi-center validation is needed. The nomogram showed good discriminative performance, however, given the modest sample size of the external validation cohort, further large-scale, multi-center prospective studies are warranted to confirm its clinical utility before implementation in routine practice.

## 5 Conclusion

This study provides suggestive evidence that TG and BMI may be associated with GDM risk, supported by both clinical and genetic evidence. A nomogram showed good and stable predictive performance in internal and external validation, suggesting its potential utility for early GDM risk stratification. However, further large-scale prospective studies are needed to confirm its clinical applicability. Bidirectional MR analysis provided suggestive evidence of a potential relationship between GDM and elevated TG levels, warranting further investigation.

## Data Availability

The raw data supporting the conclusions of this article will be made available by the authors, without undue reservation.

## Supplementary material

Supplementary Figure 1 Scatter plot of the MR analysis results on the impact of TC **(A)**, LDL-c **(B)** and HDL-c **(C)** on GDM. Each point represents a single SNP used as an IV. Each point represents a single SNP used as an IV; Slopes of the fitted lines correspond to MR estimates from different methods: IVW (light blue), MR-Egger (dark blue), simple mode (light green), weighted median (dark green) and weighted mode (red). The number of SNPs included: TC (n=191), LDL-c (n=12), HDL-c (n=7).

Supplementary Figure 2 Scatter plot of the MR analysis results on the impact of LDL-c **(A)**, HDL-c **(B)** and BMI **(C)** on GDM. Each point represents a single SNP used as an IV. Each point represents a single SNP used as an IV; Slopes of the fitted lines correspond to MR estimates from different methods: IVW (light blue), MR-Egger (dark blue), simple mode (light green), weighted median (dark green) and weighted mode (red). The number of SNPs included: LDL-c (n=5) and HDL-c (n=5).
