# Supplementary material for: Association between blood lipids, BMI and gestational diabetes mellitus in early pregnancy: a clinical study and Mendelian randomization
Source: Front Endocrinol (Lausanne). 2026 Aug 7;17:1870557. doi: 10.3389/fendo.2026.1870557 (PMC13493276; doi:10.3389/fendo.2026.1870557)
Supplement: Supplementary file 1 [file Table1.docx]

| **Supplementary Table S1.** Summary of removed SNPs in MR analysis due to palindromic with intermediate allele frequency or incompatible alleles | | | | |
| --- | --- | --- | --- | --- |
| Exposure | Outcome | nSNP | IDs of removed SNP | Reason for removal |
| **Forward MR** |  |  |  |  |
| TC | GDM | 1 | rs2001945 | Palindromic with intermediate AF |
| TG | GDM | 15 | rs11274835, rs1561748, rs28752924, rs35140741 | Incompatible alleles |
|  |  |  | rs10822163, rs11722924, rs1316753, rs17536077, rs5112, rs551243, rs5755799, rs62112763, rs6916318, rs9368503 | Palindromic with intermediate AF |
| LDL-c | GDM | 2 | rs2927485, rs449647 | Palindromic with intermediate AF |
| BMI | GDM | 58 | rs2731238 | Incompatible alleles |
|  |  |  | rs10245306, rs10423928, rs10824218, rs10898324, rs11150461, rs11152135, rs11161044, rs11161335, rs111909661, rs1126930, rs1154988, rs117176448, rs12001634, rs13264909, rs1454687, rs1608113, rs16868443, rs17132130, rs17218879, rs17744603, rs1860750, rs2133561, rs2253310, rs249293, rs2543132, rs2579040, rs2605570, rs2618039, rs28601761, rs317656, rs347551, rs3904162, rs4419475, rs4844809, rs5011579, rs56161855, rs62020775, rs62385430, rs6545144, rs6561937, rs6583310, rs6604866, rs667515, rs6749422, rs72867447, rs73141547, rs73213484, rs7323, rs7442885, rs754635, rs7568228, rs76345589, rs7761673, rs7940866, rs7946756, rs9835772, rs9860326 | Palindromic with intermediate AF |
| **Reverse MR** |  |  |  |  |
| GDM | TG | 1 | rs73410774 | Incompatible alleles |
| GDM | LDL-c | 1 | rs10830963 | Palindromic with intermediate AF |
| GDM | HDL-c | 1 | rs10830963 | Palindromic with intermediate AF |
| GDM | BMI | 1 | rs10830963 | Incompatible alleles |
| Note: AF: allele frequency. SNPs were excluded if they were palindromic with intermediate allele frequency (AF between 0.42 and 0.58) or had incompatible alleles between exposure and outcome GWAS datasets, as these issues can introduce strand ambiguity and bias in effect estimation. | | | | |
